# An Update on Endodontic Microsurgery of Mandibular Molars: A Focused Review

**DOI:** 10.3390/medicina57030270

**Published:** 2021-03-16

**Authors:** Sun Mi Jang, Euiseong Kim, Kyung-San Min

**Affiliations:** 1 Microscope Center, Department of Conservative Dentistry and Oral Science Research Center, Yonsei University College of Dentistry, Seoul 03722, Korea; sm8572@hanmail.net (S.M.J.); andyendo@yuhs.ac (E.K.); 2 Department of Conservative Dentistry, School of Dentistry, Jeonbuk National University, Jeonju 54896, Korea; 3 Research Institute of Clinical Medicine, Jeonbuk National University, Jeonju 54907, Korea; 4 Biomedical Research Institute, Jeonbuk National University Hospital, Jeonju 54907, Korea

**Keywords:** endodontic, microsurgery, mandibular, molar, update

## Abstract

Endodontic microsurgery is a highly predictable treatment option in most cases when conventional endodontic treatment is not feasible. Nevertheless, mandibular molars are still considered by clinicians to be the most difficult type of teeth, with the lowest success rate. In recent years, endodontic microsurgery has been attempted more frequently with the emergence of modern cutting-edge technologies such as dental operating microscopes, various microsurgical instruments, and biocompatible materials, and the success rate is increasing. This review describes the current state of the art in endodontic microsurgical techniques and concepts for mandibular molars. Notably, this review highlights contemporary equipment, technology, and materials.

## 1. Introduction

Endodontic treatment aims to disinfect the root canal system, followed by sealing this space to prevent recontamination [1]. For this purpose, nonsurgical endodontic treatment is the first option in most cases, but a surgical approach may be indicated when peri-radicular pathosis cannot be solved by a nonsurgical method [2]. Surgical endodontic treatment has evolved considerably owing to new technological advances such as dental operating microscopes, cone-beam computed tomography (CBCT), computer-aided design/computer-aided manufacturing (CAD/CAM), and three-dimensional printing technology. Most endodontists now consider the above equipment essential for surgical endodontic treatment.

Nevertheless, clinicians still consider mandibular molars to be the most difficult targets. The success rate for mandibular molars is lower than that for any other type of teeth in the oral cavity [3]. The difficulty posed by mandibular molars is their anatomical location in the posterior areas of the oral cavity. Even in a retrospective study that used a 15-year database obtained in the 21st century, molars showed a higher probability of failure than anterior teeth, and tooth type might be the only factor contributing to the outcomes of microsurgery [4]. Therefore, microsurgery of mandibular molars requires an adequate evaluation of the surgical access and the relationship of the roots to anatomical structures such as the mental foramen and mandibular canal [5].

However, within the limitation of our knowledge, there has been no focused review regarding endodontic microsurgery of mandibular molars in the past 10 years, although considerable technical advances have occurred. Therefore, the purpose of this review was to present an update on endodontic microsurgical techniques and concepts for mandibular molars.

## 2. Anatomical Considerations

### 2.1. Thickness of the Buccal Bone

**Figure 1:**
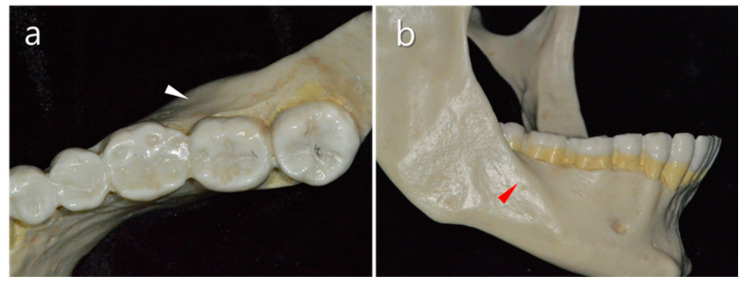
Anatomical features of the mandibular molar area. (**a**) Thick buccal bone of the mandible (white triangle). (**b**) Oblique ridge in the second mandibular molar area (red triangle).

The buccal bone of the mandibular molar area is thicker than in other areas. The thickness of the posterior buccal cortical plate is generally 2–3 mm on average (Figure 1a). Furthermore, this thickness may increase posteriorly (oblique ridge), limiting access to the molar roots (Figure 1b). While the mesial root of the mandibular first molar is reasonably close to the buccal cortical plate, the distal root is centrally located in the bone and the roots of the second molar are significantly closer to the lingual cortical plate [6]. Therefore, mandibular molars with a thick buccal bone plate pose a challenging problem in endodontic surgery, especially for precise root-end localization.

**Figure 2:**
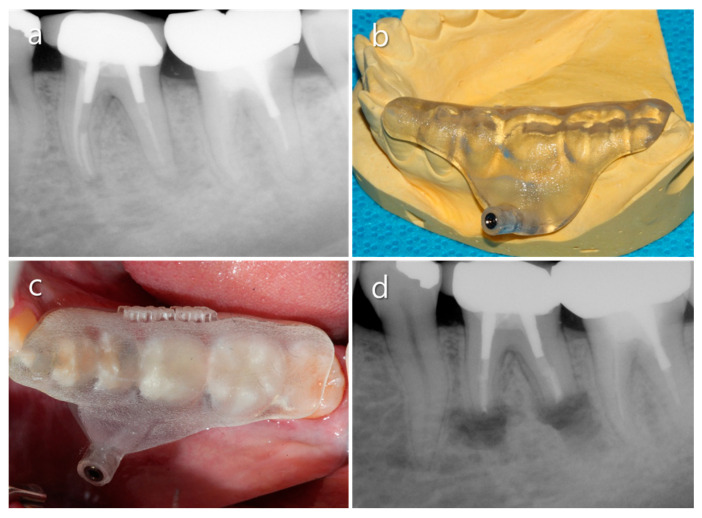
Clinical case using a computer-aided design/computer-aided manufacturing (CAD/CAM)-guided surgical template to localize the root apex through thick. (**a**) Diagnostic periapical X-ray. (**b**) A manufactured surgical template. (**c**) The surgical template was positioned on the tooth. (**d**) Postoperative radiograph.

Recently, Bi et al. [7] reported a sophisticated approach that reduced the distance between the roots and the buccal cortical plate by orthodontic movement. By orthodontic movement toward the buccal aspect for 2 months, the distance between the two anatomical structures was decreased from 4.5 mm to 1.5 mm (mesial root) and 7.1 mm to 4.5 mm (distal root). Therefore, the surgical procedure became easier and the results were successful. Furthermore, Ahn et al. [8] performed osteotomy and apex localization effectively using a CAD/CAM-guided surgical template in a mandibular molar with a thick buccal bone plate (Figure 2). Indeed, the surgical template manufactured by using CAD/CAM technology combined with CBCT can determine the three-dimensional extent of the required cortical bony window. Therefore, with this guide, the surgical approach to the apical region is less invasive and less traumatic so that minimizes bone loss in comparison to the traditional osteotomy techniques [9]. However, even with this static technique, clinicians may have problems regarding the instrumentation during the surgical procedure. In this respect, most recently, dynamic navigation systems for dental implant positioning were applied to endodontic microsurgery [10]. This dynamic navigation integrates surgical procedure and radiologic images by using an optical positioning device controlled by a dedicated computerized interface. Consequently, with the help of cutting-edge guiding technologies, clinicians can perform a safe and less traumatic surgical procedure in the mandibular molar area, and unfavorable complications including increased postoperative pain, delayed healing, and nerve damage can be avoided.

### 2.2. Vestibular Fornix

**Figure 3:**
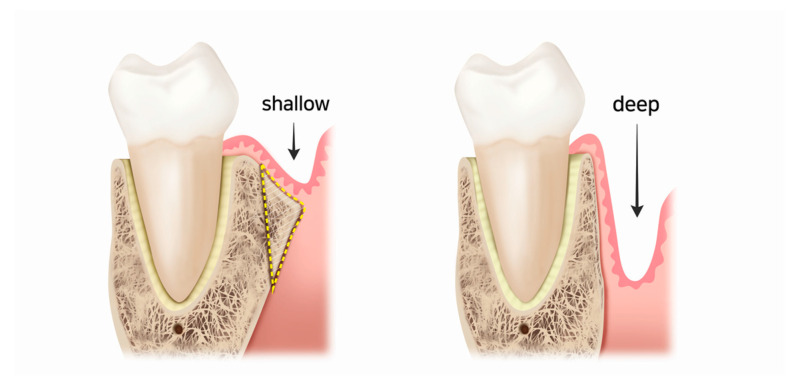
Drawings of mandibular molars and their mesiodistal supporting structures. The vestibular fornix becomes deeper by reducing the buccal cortical plate.

The depth of the vestibular fornix is considered a reliable predictor of the possible difficulty that may be encountered during periapical surgery of the mandibular posterior teeth [11]. If the vestibular fornix is shallow, the buccal alveolar bone overlying the roots will be thick and consequently access to the root end will be limited. In some cases, therefore, the buccal cortical plate can be reduced before exploring for the periapical lesion or root end (Figure 3). In this procedure, the surgical concept of vestibuloplasty can be applied. However, clinicians should bear in mind that this procedure may destroy a considerable amount of bone. Instead of endodontic surgery, extraction and replantation/dental implantation can be a less traumatic alternative, especially for mandibular second molars.

### 2.3. Mandibular Canal

**Figure 4:**
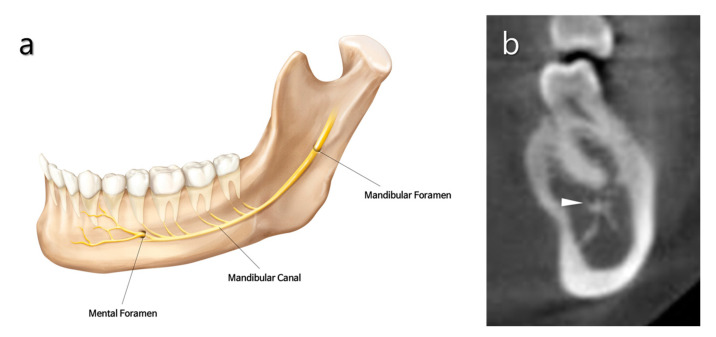
Anatomical features of the mandibular canal and mental foramen. (**a**) Schematic illustration of human mandible showing the innervation of mandibular and mental nerves. (**b**) Transverse section of a cone-beam compute tomography (CBCT) scan through the mandible in the region of the root of the first molar. The mandibular canal (white triangle) is closely located lingual to the root apex.

The mandibular canal is sometimes in the proximity of the roots of all mandibular molars (Figure 4a). According to a systematic review, the shortest distances from the superior cortical bone of the mandibular canal to the first and second molars were 3.82 mm and 1.4 mm, respectively [12]. Even a direct contact relationship was found in 3.3% of the first molar and 16% of the second molar [13]. Furthermore, the distance from the root to the canal was shorter in younger people (<21-year-old) than in older people [13,14]. Anatomically, the mandibular canal is situated lingually to the roots of the mandibular first molars (Figure 4b).

**Figure 5:**
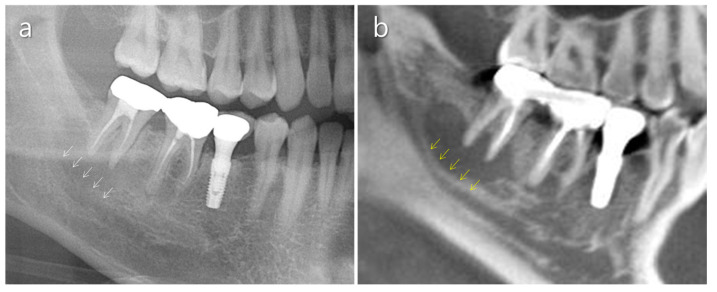
Usefulness of CBCT for identifying the mandibular canal. (**a**) Unclear mandibular canal border in a conventional panoramic radiograph. Periapical bone resorption adjacent to the mandibular canal makes identification of the upper border of the canal difficult (white arrows). (**b**) A CBCT scan shows the upper border of the mandibular canal remarkably well (yellow arrows).

The borders of the mandibular canal are often difficult to visualize with conventional radiographic techniques (Figure 5a). A parallel periapical radiograph, either horizontally or vertically positioned, can usually provide a reasonably accurate image of the relationship between the superior border of the mandibular canal and the root apices. CBCT imaging can be very useful for identifying the location of the mandibular canal and determining its relationship to the root apices (Figure 5b) [15]. Martí et al. [16] reported that periapical surgery is a valid treatment option for mandibular molars, even those situated in close proximity to the mandibular canal (less than 2 mm).

### 2.4. Mental Nerve

**Figure 6:**
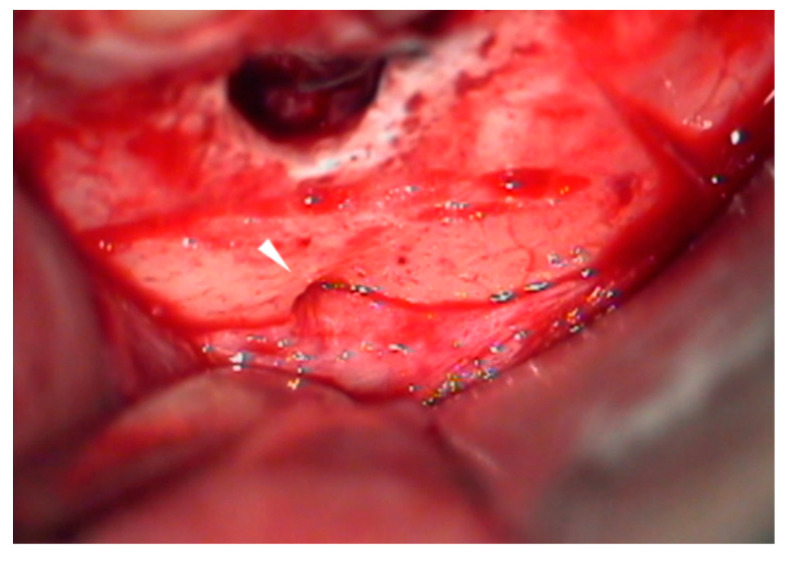
Clinical features of the mental foramen and nerve bundle (white triangle) that are detected during the surgical procedure.

The mental nerve, a branch of the inferior alveolar nerve, emerges with the mental artery through the mental foramen to supply the oral mucosa and the skin of the lower lip and chin (Figure 6). It can be damaged during flap retraction, and paresthesia may occur. The mental foramen usually appears on a radiograph as a round radiolucent area below the mandibular second premolar root apex. However, its location varies and it may be seen at the same level or superior to the root apices [11].

The mental foramen is most usually single in humans; when it is double or multiple, the additional foramen is termed an accessory mental foramen. Accessory mental foramina are reported to be rare, with a prevalence ranging from 1.4% to 10% [17,18,19,20]. Moreover, Iwanaga et al. [21] reported that accessory mental foramina smaller than 1.3 mm^2^ were not clearly identified on surface-rendered CBCT images. In this respect, paresthesia may occur even if the clinician does not recognize the damage to the mental nerve [22,23].

**Figure 7:**
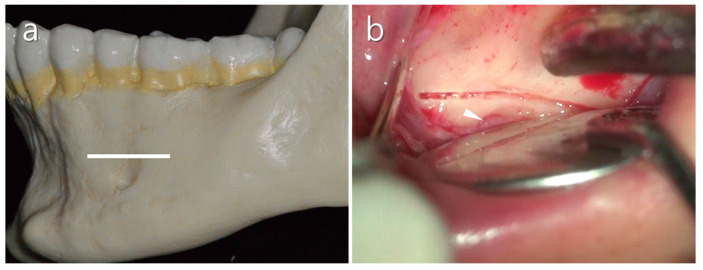
The groove technique. (**a**) A narrow horizontal groove is positioned just above the mental foramen (white line). (**b**) Clinical feature of a practically narrow groove made using the piezoelectric saw. Note that the nerve/vessel bundle comes from the foramen (white arrow).

Gutmann and Harrison [5] suggested protecting the mental nerve by positioning the periosteal retractor between the mental foramen and the intraosseous surgical site. More recently, Kim and Kratchman [24] suggested an effective tip, the “groove technique”, to prevent mental nerve damage. Briefly, a 15-mm-long horizontal groove is prepared beyond the root apex and the retractor is positioned within the groove (Figure 7a). This technique permits the secure retraction of the flap protecting the nerve during the procedure. Recently, the use of a piezoelectric saw instead of a bur has been shown to be useful to obtain a groove as narrow as is practical for the placement of the retractor (Figure 7b). By using this method, excessive bone removal can be avoided.

### 2.5. Distolingual Root

Mandibular first permanent molars usually have two roots located mesially and distally, but in Asian populations, an additional distolingual root is considered a normal morphological variant [25,26]. In two studies conducted in Korea, the prevalence of independent distolingual roots was 24.5% and 22.8%, respectively [27,28]. These separate distolingual roots of mandibular first molars are considered too difficult for performing periapical surgery due to the considerable distance from the buccal cortical plate (8.63 mm) [28].

Due to this difficulty, few case reports have described surgical endodontic management of these roots. To our knowledge, only one report was found [29], and the authors performed a reduced amount (2 mm) of root resection and root-end preparation and suggested that distolingual roots can be successfully treated by modifying the standard protocol. Nevertheless, periapical surgery for the distolingual root should be undertaken with careful deliberation due to the technical difficulty.

### 2.6. Mandibular Second Molars

**Figure 8:**
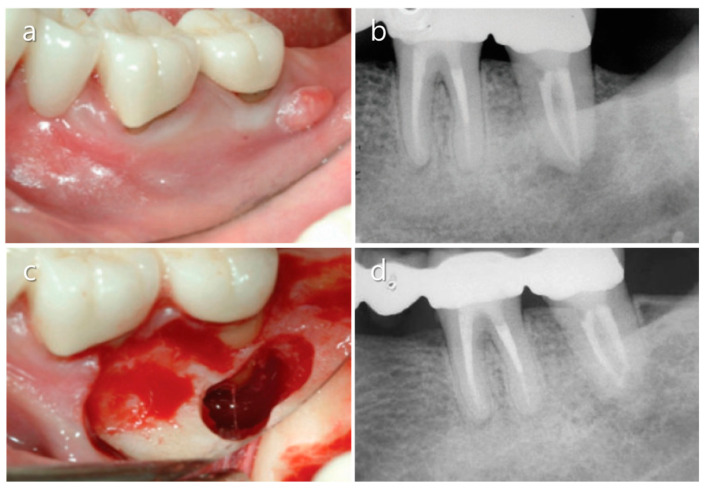
Endodontic surgery of a mandibular second molar. (**a**) Sinus tract formed on the buccal aspect of a left mandibular second molar. (**b**) Diagnostic periapical X-ray image showing that the tooth was an abutment for a fixed prosthodontic restoration. (**c**) The root apex was exposed after osteotomy. (**d**) A five-year follow-up radiograph shows complete bony healing. Images were modified from Song [31] after receiving permission from the journal.

Due to their location, endodontic microsurgery for mandibular second molars is seldom attempted even by endodontic specialists. Access to the root apex of the teeth is usually obtained by intentional replantation since the roots often have a convergent form. Nevertheless, a surgical approach for mandibular second molars is attempted in certain situations, such as abutment of fixed prosthodontics (Figure 8). In endodontic surgery for second molars, the position of the mandibular canal should be considered carefully. The mandibular canal is located buccal to the apex of the second molar, but lingual to the apex of the first molar in most cases [30]. Furthermore, it was reported that the second molars had a close distance to the canal (mean, 3.7 mm).

## 3. Surgical Considerations

### 3.1. Flap Design

**Figure 9:**
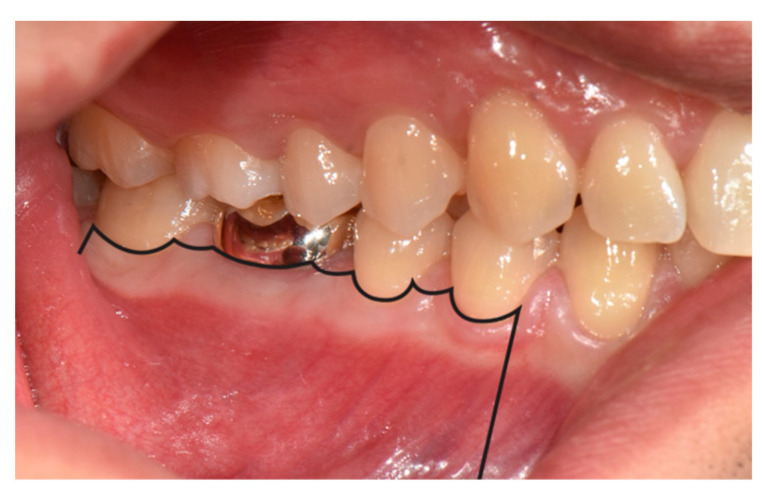
Papilla base incision (black line), creating access to the mandibular first molar.

Flap design is of critical importance in endodontic surgery. It affects access, visibility, anatomical structures, repositioning, and suturing [5]. The triangular full-thickness flap with intrasulcular incision has been widely used in periapical surgery for the mandibular molar area. However, this incision results in recession and shrinkage of the papilla [32]. In this respect, currently, the papilla base incision (PBI) is recommended to prevent loss of interdental papilla height (Figure 9). In the study by Velvart et al. [33], no recession was found when the healing of the PBI was examined after one year. In another study comparing PBI and intrasulcular incision, the gingival margin remained higher when a PBI was performed [34]. For this reason, the popular semilunar and Luebke-Ochsenbein flap designs are no longer recommended [24].

The vertical incision should be made parallel to the vessels in the attached gingiva and submucosa [35]. Traditionally, angulation of the vertical incision to create a broad-based flap, with the vestibular portion of the flap wider than the free end, has been suggested [36]. This design was based on the expectation of improvement of blood supply to the flap, but this idea is no longer supported scientifically. It created a lasting scar by cutting the mucosal tissue across the fiber lines [37].

**Figure 10:**
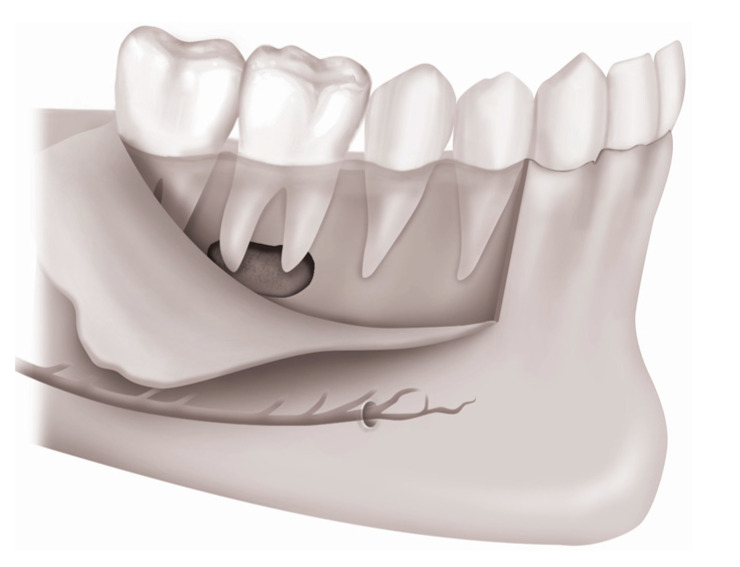
Vertical incision for endodontic microsurgery of the mandibular first molar. Note that the vertical incision was placed on the mesial line angle of the mandibular first premolar and parallel to the root longitudinally. This helps to avoid mental nerve injury and the scar on the gingival tissue.

A vertical incision is usually recommended only on the mesial side of the planned molar. The rectangular flap design with two vertical incisions is limited in the posterior portion of the mandible by anatomical considerations. A distal vertical incision provides no advantages, but causes suturing problems due to the extremely limited space in that area. When the first molar is treated, the vertical incision is commonly placed on the mesial line angle of the first premolar (Figure 10). Consequently, an incision at this location does not damage the mental foramen and muscle attachment located around the second premolar. On the contrary, Moiseiwitsch [38] suggested placing the vertical incision distal to the surgical site to avoid damage to the mental nerve. However, this approach has not been mentioned further in the literature.

### 3.2. Osteotomy

**Figure 11:**
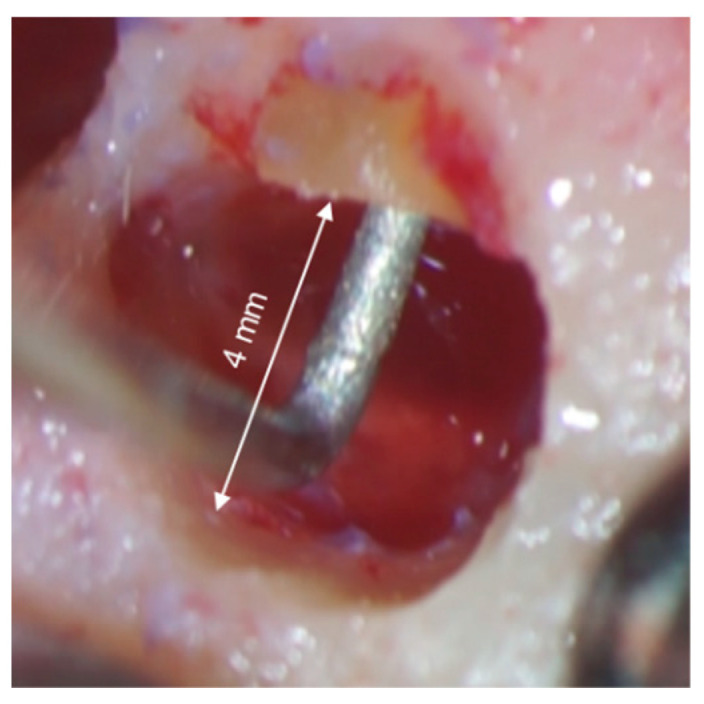
Bone crypt (4 mm in diameter) after osteotomy, allowing an ultrasonic tip to move without interference.

Evidence has accumulated showing that the larger the defect, the smaller the chance that complete healing will occur. Furthermore, Rubinstein and Kim [39] showed a direct relationship between the size of osteotomy and the speed of healing: the smaller the osteotomy, the faster the healing. Indeed, in microsurgery of the mandibular first molar, osteotomy of 3 to 4 mm in diameter suffices, just enough to provide space for a 3-mm ultrasonic tip (Figure 11).

**Figure 12:**
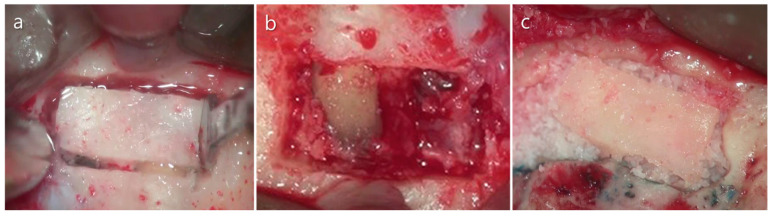
The “bony window technique” with a piezoelectric saw. (**a**) Bone preparation for the cortical window. (**b**) Uncovered area of the lesion and the apices of the roots. (**c**) Allogenic bone was grafted and cortical bone was repositioned. Images were modified from Kim et al. [40] after receiving permission from the journal.

Recently, the preservation of the cortical bony plate, the so-called “bone window technique”, has been attempted using a piezoelectric saw (Figure 12). With this conservative technique, the removed cortical bony plate can be repositioned onto the original site. Piezoelectric instruments ensure precise and safe selective sectioning of bone, and the air-water cavitation effect provides better visibility by creating a clear and blood-free surgical field [40]. With these properties, osteotomy can be performed safely, less traumatically, and more conveniently, especially in the mandibular molar area. In a preliminary study, patients who underwent a conventional osteotomy in their mandibular molar area presented more swelling than those who were treated with a bony window technique [41]. Recently, Lee et al. [42] reported a case of periapical surgery on the mandibular first molar and showed that the repositioned cortical bony plate acted as an autologous graft for the surgical site, thereby minimizing bone loss. Furthermore, this technique can be facilitated by using the aforementioned CAD/CAM-aided guiding template [43]. Kulakov et al. [44] performed 11 cases of piezoelectric surgery on the mandibular molars with repositioning of the cortical bone block, and the repositioning procedure was considered a highly acceptable technique.

### 3.3. Root-End Resection

**Figure 13:**
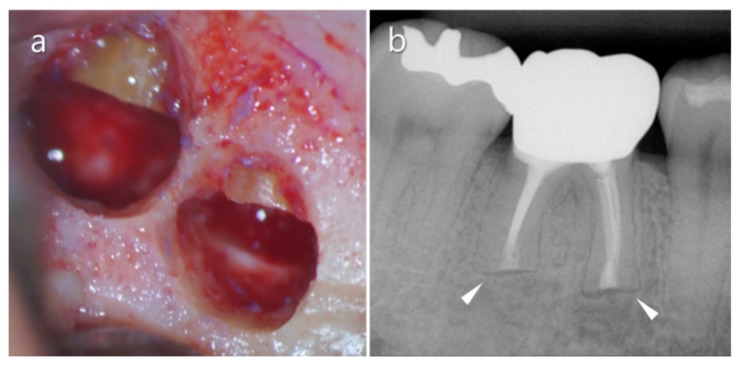
A contemporary technique for root resection. (**a**) Clinical features of mesial and distal roots resected perpendicular to the long axis of each root. (**b**) A periapical X-ray image taken using the parallel technique shows resection with a shallow bevel. Note that complete bony healing occurred with continuous lamina dura.

A consensus has been reached regarding the amount of root-end resection. It is recommended that 3 mm should be resected since most of the ramifications/lateral canals that may serve as a microbial harbor are thereby removed. Furthermore, the modern technique uses a shallow bevel angle of 0°–10° to expose fewer dentinal tubules with enhanced magnification and illumination techniques [24] (Figure 13).

**Figure 14:**
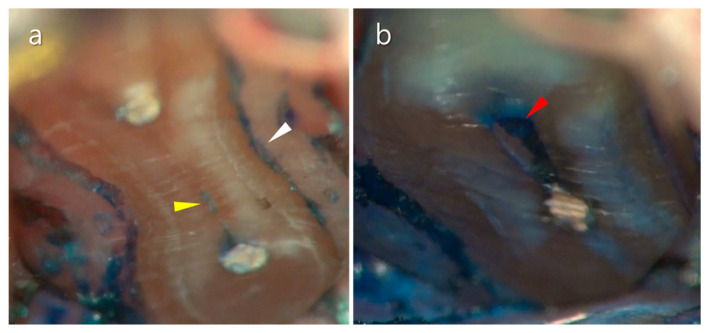
A resected root surface stained with methylene blue solution. (**a**) The periodontal ligament was stained and clearly distinguished from the adjacent bone (white triangle). The isthmus between the mesio-buccal and mesio-lingual canal was also stained (yellow triangle). (**b**) The stained remnant was identified in the unfilled distal root canal (red triangle).

Resection of the entire root end in mandibular molars is challenging. In addition to the thick cortical bone, the cross-sectional morphology of the root apex of the mandibular molar is broad buccolingually. Therefore, careful inspection of the root end is mandatory to verify whether it is completely resected. For this purpose, methylene blue solution is helpful for differentiating the root end from adjacent bone tissue by staining the periodontal ligament space (Figure 14a). Sometimes, using a long shank bur is necessary for complete resection of the root end. In some cases, the remaining remnants in unfilled root canals can be identified by methylene blue staining under high magnification (Figure 14b).

### 3.4. Root-End Preparation

**Figure 15:**
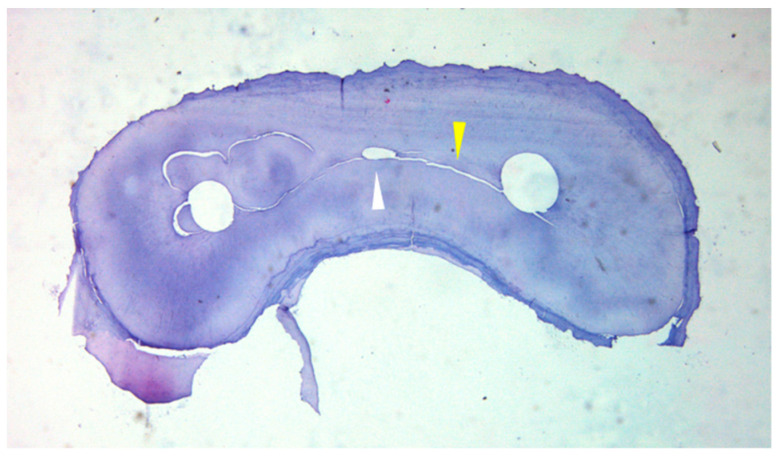
Histologically sectioned mesial root of the mandibular first molar at 3 mm level from the apex after canal shaping. The white triangle indicates an unprepared middle mesial root. The yellow triangle indicates the isthmus connecting the canals.

**Figure 16:**
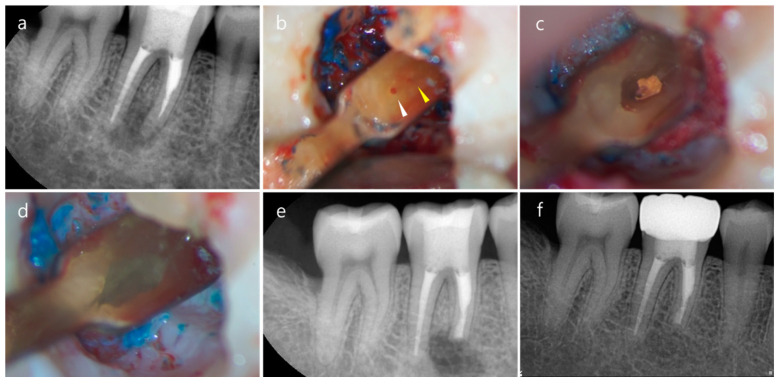
Endodontic microsurgery on an isthmus-absent mandibular molar. (**a**) A preoperative periapical radiograph shows periapical radiolucency and unsatisfactory canal filling on the mesial root due to an unnegotiable ledge. (**b**) The resected root surface shows mesio-buccal (white triangle) and mesio-lingual (yellow triangle) canals, which are closely located without an isthmus at 3 mm from the apex. (**c**) The simple, oval-shaped root-end cavity. (**d**) The root-end filling with mineral trioxide aggregate (MTA). (**e**) A postoperative periapical radiograph. (**f**) A 15-month follow-up radiograph shows that complete healing occurred.

Mandibular molars have an elaborate root canal anatomy, particularly in the apical portion. Mesial roots usually have two canals (buccal and lingual) and they are connected with the isthmus. In mandibular molars, an isthmus is frequently observed in sections between 3 and 4 mm from the apex (Figure 15). Karunakaran et al. [45] reported that the prevalence of isthmuses in the mesial roots of mandibular first molars was 97.2%, while the prevalence in distal roots was 39%. Furthermore, von Arx [46] analyzed the occurrence of a canal isthmus in mandibular molars during endodontic surgery. He reported that 83% of mesial roots had two canals with an isthmus and that 36% of distal roots had two canals with an isthmus. Recently, Kang et al. [47] inspected 31 mesial roots of mandibular first molars and found that 100% of roots had an isthmus at 3 mm level from the apex. Predictably, the success rate for endodontic microsurgery on isthmus-absent teeth was higher than that for isthmus-present teeth [48], which might be due to the difference in the level of difficulty for identification and instrumentation. In this respect, it is strongly recommended to identify and prepare the isthmus as well as root canals. On the contrary, if an isthmus is not present, a simple oval-shaped root-end cavity suffices for obturation (Figure 16). Similar to periodontal ligament identification, methylene blue is the most effective way to stain an isthmus during the surgical procedure (Figure 14a). Furthermore, it is essential that the isthmus should be prepared to a depth of 3 mm like the root canals.

**Figure 17:**
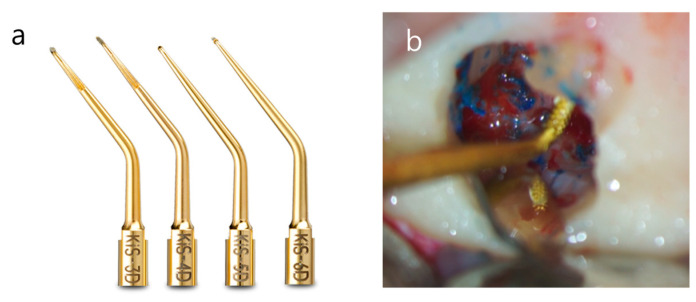
(**a**) Diamond-coated ultrasonic tips with various angles (Microsurgical KiS tips). 3D and 5D for buccal canals. 4D and 6D for lingual canals. (Image courtesy of Young Specialties, https://www.youngspecialties.com/product/kis-ultrasonic-tips-1-ct, accessed on 27 January 2021) (**b**) Retrograde cavity preparation for mesial root-end of a mandibular first molar with JETip.

Ultrasonic instruments are used for root-end cavity preparation. Smooth stainless steel was used in the early days, but diamond-coated instruments (e.g., KiS tips; Young Specialties, Algonquin, IL, USA) are now highly recommended since the coated tips cut the dentin and filling material much faster than stainless steel [49,50] (Figure 17a). Notably, these instruments are advantageous for multi-rooted mandibular molars, especially when an isthmus exists. Most recently, ultrasonic tips with numerous metal micro-projections on the cutting surface instead of diamond coating are also available (JETip; B&L Biotech, Fairfax, VA, USA) (Figure 17b).

The most important aspect of mandibular molar surgery is the angle of the instrument. Double-angled instruments are strongly recommended for mandibular molars since the accessibility is much better compared to single-angled general tips. Manufacturers provide various double-angled tips suitable for buccal and lingual canals (e.g., KiS 3D and 5D for buccal canals, KiS 4D and 6D for lingual canals).

### 3.5. Root-End Filling

An ultrasonically prepared root-end cavity must be filled with a material that guarantees a bacteria-tight seal. Various materials have been suggested for this purpose. Amalgam has been used as a retrograde filling material for many years, but the efficacy of amalgam has been questioned due to unfavorable physical properties and environmental damage [51]. More recently, zinc oxide eugenol (ZOE) containing materials such as intermediate restorative material (IRM) and super ethoxy-benzoid acid (Super-EBA) has been widely used due to its superior sealability compared to amalgam [52]. However, shortcomings of the ZOE-containing materials are their toxicity and susceptibility to moisture. Finally, calcium silicate cement, widely known as mineral trioxide aggregate (MTA), has gained considerable attention for the past quarter of a century due to its favorable sealing ability and biocompatibility [53]. Notably, this hydrophilic material sets in an aqueous environment [54]. However, the main disadvantage of MTA is its poor handling properties [55]. This is an obstacle regarding the application of MTA, particularly in the mandibular molar area, which has limited access and visibility. In this respect, efforts have been made to overcome this problem by enhancing the application method or handling ability of the materials.

**Figure 18:**
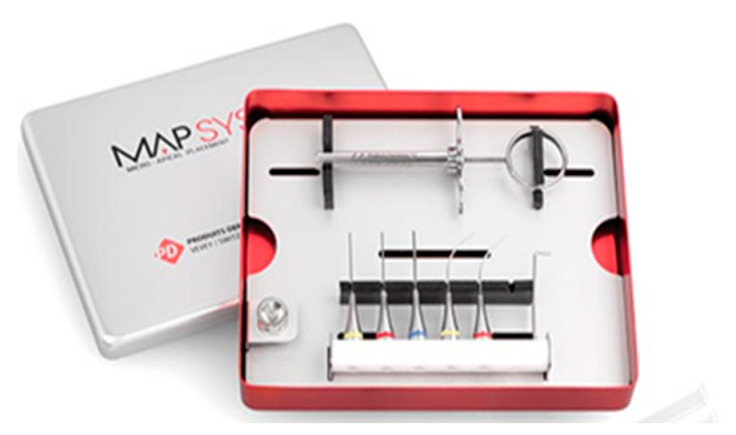
MAP system universal kit. (Image courtesy of Dentsply Sirona, https://www.maillefer.com/products/specialty/map-system, accessed on 27 January 2021).

Several instruments have been developed to facilitate the application of MTA. The first one that became available was the Dovgan Carrier, which was designed specifically for the delivery of MTA by Joseph Dovgan. It features a NiTi plunger as well as a removable luer-lock lumen that can be removed for cleaning or replacement if it is damaged or clogged. It offers the choice of three different tip diameters. Similar instruments were then launched by several manufacturers such as the Micro Apical Placement (MAP) system (Dentsply-Maillefer, Ballaigues, Switzerland) (Figure 18). Notably, the “needles” with triple angles of the MAP instrument are designed to place MTA in the root-end cavity in the most difficult anatomical regions such as the mandibular molars [56]. However, if mixed MTA is left within the carrier after the appointment, the tip may become clogged and seemingly unusable. Therefore, careful management of the instruments is required.

**Figure 19:**
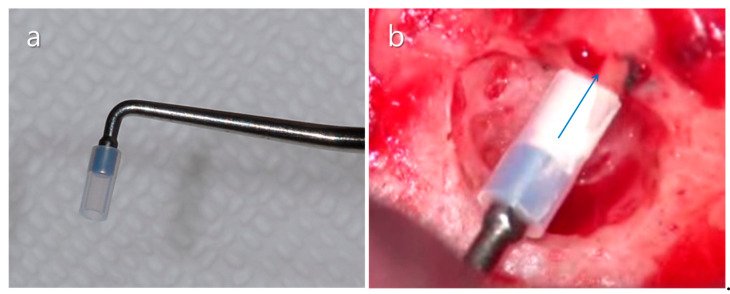
Manual carrier for MTA application. (**a**) Assembled Teflon sleeve and plugger. (**b**) Clinical application of MTA into root-end cavity prepared in the buccal root of the mandibular first molar using the manual carrier. A blue arrow indicates the direction of the force of the plugger.

Another type of manual carrier, which consists of a disposable Teflon sleeve and plugger, is available in the endodontic market (Dentsply Sirona) (Figure 19a). This instrument is also useful for placing MTA in the root-end cavity of mandibular molars because clinicians can push a relatively large amount of MTA into the cavity with smooth pressure (Figure 19b). However, the use of the manual carrier is limited when the bony cavity is small and the roots are located deep in the buccal bony plate since the vertical dimension of the plugger is relatively long.

**Figure 20:**
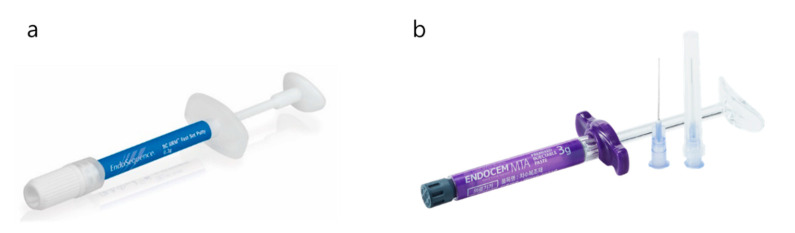
Commercially available premixed calcium silicate root-end filling materials. (**a**) Endo-sequence Root Repair Material (RRM). (Image courtesy of Brasseler, https://brasselerusadental.com/products/bc-rrm-fast-set-putty/, accessed on 28 January 2021) (**b**) Endocem MTA Premix (Image courtesy of Maruchi).

Several attempts have also been made to enhance the handling ability by modifying the material properties. Biodentine (Septodont, St. Maurdes Fossés, France) is a moldable MTA-based material that contains a hydro-soluble polymer in liquid, which reduces the viscosity of the cement and thereby improves handling [57]. Therefore, Biodentine can be applied easily with conventional instruments such as stoppers, without the aforementioned specially designed instruments. Furthermore, injectable bio-ceramic root-end filling materials have also been developed (Figure 20). They are premixed with polymeric vehicles and preserved in water-tight syringes so that the materials can be placed directly by injection. They are also moldable and can be delivered by simple instruments. Consequently, the materials can be placed more conveniently in the mandibular molar area.

## 4. Conclusions

Endodontic microsurgery for the mandibular molar area is challenging for clinicians due to the difficulty in access and the critical anatomical structures. Owing to the development of cutting-edge technology including equipment and materials, the success rate has been increasing. Therefore, clinicians should be familiar with up-to-date information on the current concepts to achieve successful outcomes, and this review can be a useful guideline for the preservation of mandibular molars through microsurgery.
